# Supplementary material for: Interface-induced sign reversal of the anomalous Hall effect in magnetic topological insulator heterostructures
Source: Nat Commun. 2021 Jan 4;12:79. doi: 10.1038/s41467-020-20349-z (PMC7782489; doi:10.1038/s41467-020-20349-z)
Supplement: Supplementary file 1 — Supplementary Information [file 41467_2020_20349_MOESM1_ESM.pdf]

Supplementary Information

Interface-Induced Sign Reversal of the Anomalous Hall Effect in Magnetic Topological Insulator Heterostructures

Fei Wang[1, 2, 3, 5], Xuepeng Wang[4,5], Yi-Fan Zhao[1,5], Di Xiao[1], Ling-Jie Zhou[1], Wei Liu[2], Zhidong Zhang[2], Weiwei Zhao[3], Moses H. W. Chan[1], Nitin Samarth[1], Chaoxing Liu[1], Haijun Zhang[4], and Cui-Zu Chang[1]

[1]Department of Physics, The Pennsylvania State University, University Park, PA 16802, USA

[2]Shenyang National Laboratory for Materials Science, Institute of Metal Research, Chinese Academy of Sciences, Shenyang 110016, China

[3]School of Material Science and Engineering, Harbin Institute of Technology, Shenzhen, 518055, China

[4]National Laboratory of Solid-State Microstructures, School of Physics, Nanjing University, Nanjing 210093, China

[5]These authors contributed equally to this work.

Corresponding authors: zhanghj@nju.edu.cn (H. Z.); cxc955@psu.edu (C.-Z. C.).

Supplementary Note 1. Sample structures and sample characterizations

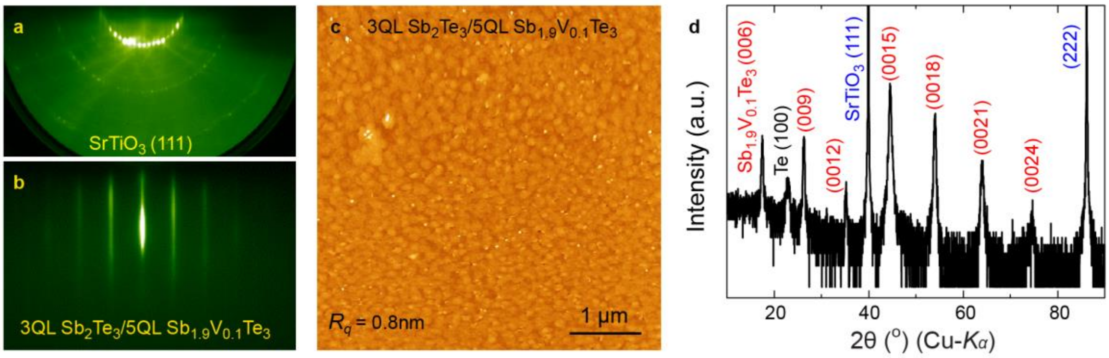

Supplementary Fig. 1 | Characterizations of magnetic TI heterostructures. (a,b) RHEED patterns of the heat-treated $SrTiO_3$ (111) substrate (a) and the 3 QL Sb_2Te_3/5 QL $Sb_{1.9}V_{0.1}Te_3$ heterostructure (b). (c) AFM image of the 3 QL Sb_2Te_3/5 QL $Sb_{1.9}V_{0.1}Te_3$ heterostructure. (d) XRD spectrum of the 10 nm Te capped 20 QL $Sb_{1.9}V_{0.1}Te_3$ sample.

Supplementary Figs. 1a and **1b** display the reflection high energy electron diffraction (RHEED) patterns of the heat-treated insulating $SrTiO_3$ (111) substrate and the 3 QL Sb_2Te_3/5 QL $Sb_{1.9}V_{0.1}Te_3$ film, respectively. The clear reconstruction (**Supplementary Fig. 1a**) indicates the flat surface of the heat-treated $SrTiO_3$ (111), which makes it suitable for the molecular beam epitaxy (MBE) growth of the topological insulator (TI) films/heterostructures. The streaky "1×1" patterns of the 3 QL Sb_2Te_3/5 QL $Sb_{1.9}V_{0.1}Te_3$ bilayer reveal its highly-ordered crystalline structure (**Supplementary Fig. 1b**). The flat surface of the 3 QL Sb_2Te_3/5 QL $Sb_{1.9}V_{0.1}Te_3$ heterostructure is also seen in an atomic force microscopy (AFM) image (**Supplementary Fig. 1c**). The root-mean-squared roughness (R_q) of this heterostructure sample is ~ 0.8 nm over a 5 μm × 5 μm scanning area. **Supplementary Fig. 1d** shows the X-ray diffraction (XRD) spectrum of the 10 nm Te capped 20 QL $Sb_{1.9}V_{0.1}Te_3$ sample, the sharp (00*n*) peaks observed here further demonstrates the high crystalline property of the magnetic TI films.

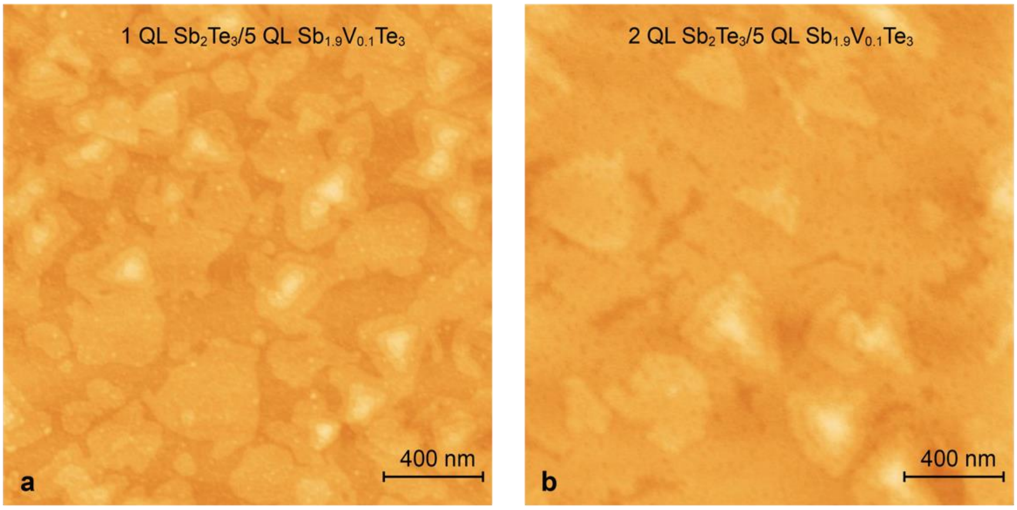

Supplementary Fig. 2 | AFM images of the TI/magnetic TI heterostructures. (a) AFM images of the m = 1 sample on $SrTiO_3$ (111) substrate, with R_q ~0.60 nm. (b) AFM images of the m = 2 sample on $SrTiO_3$ (111) substrate, with R_q ~0.64 nm.

We also carried out atomic force microscopy (AFM) on the m = 1 and m = 2 samples. Both samples exhibit similar morphology with R_q ~0.60 nm and ~0.64 nm, respectively (**Supplementary Fig. 2**). We noted that 1 QL holes and islands indeed exist on the surface of these two samples, but the total area of 1 QL holes and islands is less than 20%. All behaviors agree well with prior studies[1, 2].

Supplementary Note 2. Transport results of V-doped Sb_2Te_3 films

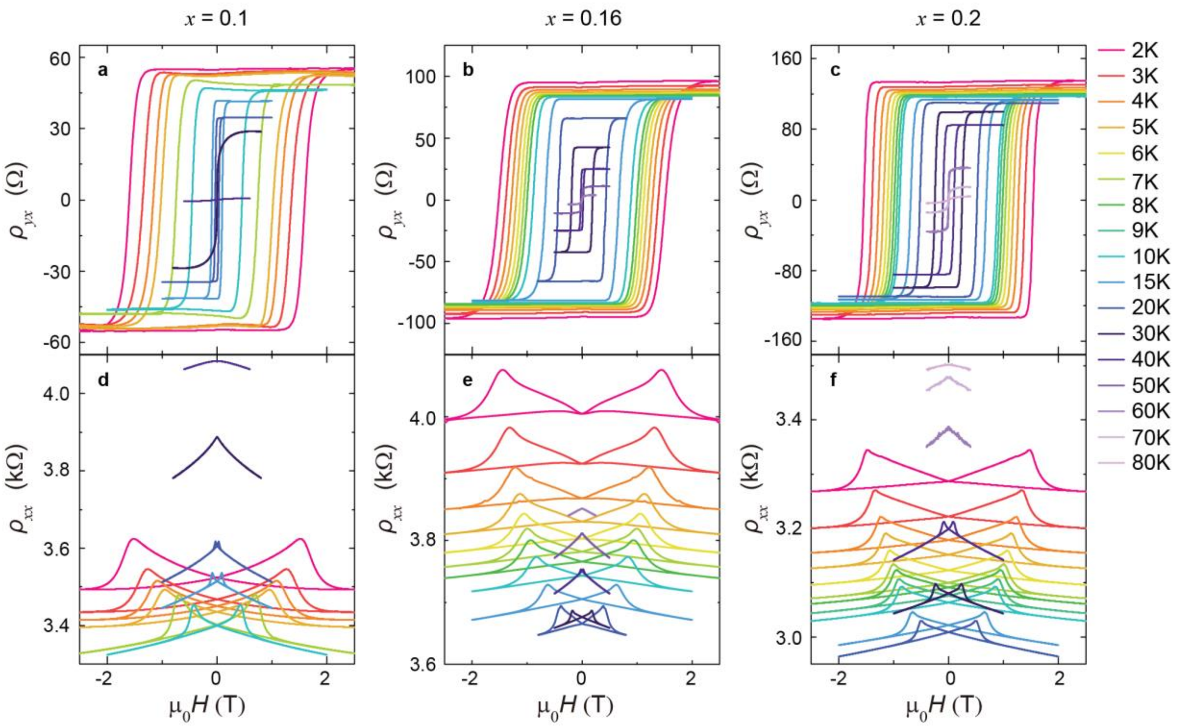

Supplementary Fig. 3| Hall traces and magnetoresistance of 5 QL $Sb_{2-x}V_xTe_3$ films at different temperatures. (a-c) Magnetic field $\mu_0 H$ dependence of the anomalous Hall resistance ρ_{yx} of 5QL $Sb_{2-x}V_xTe_3$ films with x = 0.1 (a), x = 0.16 (b), and x = 0.2 (c). (d-f) $\mu_0 H$ dependence of the longitudinal resistance ρ_{xx} of 5QL $Sb_{2-x}V_xTe_3$ films with x = 0.1 (d), x = 0.16 (e), and x = 0.2 (f).

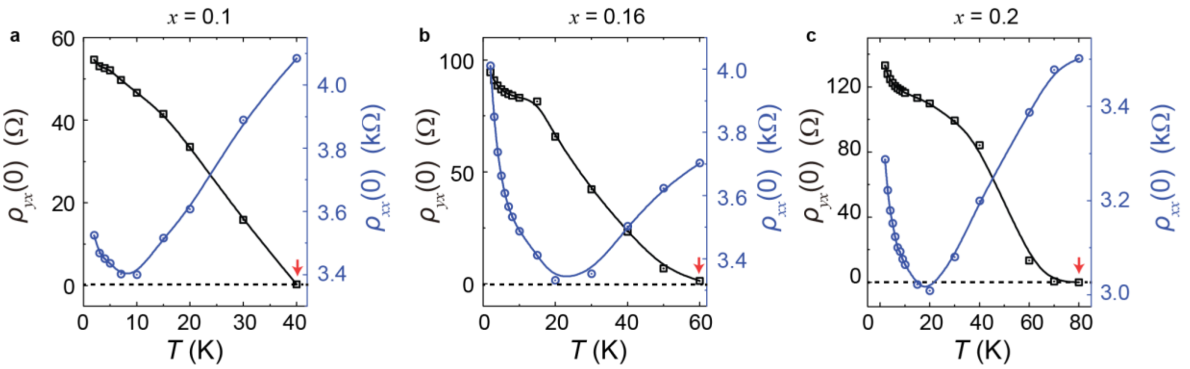

Supplementary Fig. 4| Temperature dependence of the zero magnetic field Hall resistance $\rho_{yx}(0)$ and longitudinal resistance $\rho_{xx}(0)$ of 5 QL $Sb_{2-x}V_xTe_3$ films. (a) x = 0.1, (b) x = 0.16, and (c) x = 0.2.

Supplementary Figs. 3a to **3f** show magnetic field $\mu_0 H$ dependence of the anomalous Hall (AH) resistance ρ_{yx} and the longitudinal resistance ρ_{xx} of the $Sb_{2-x}V_xTe_3$ films with different doping concentration x. At $T = 2$ K, the Hall traces show a square-shaped hysteresis loop and the corresponding magnetoresistances (MR) exhibit a butterfly feature, confirming the long-range ferromagnetic order with perpendicular anisotropy in $Sb_{2-x}V_xTe_3$ samples. The temperature dependence of the zero magnetic field Hall resistance $\rho_{yx}(0)$ and the zero magnetic field longitudinal resistance $\rho_{xx}(0)$ results are summarized in **Supplementary Fig. 4**. $\rho_{yx}(0)$ decreases with increasing temperature and vanishes at $T = 40$ K, 60 K, and 80 K for $x = 0.1$ (**Supplementary Fig. 4a**), 0.16 (**Supplementary Fig. 4b**), and 0.2 (**Supplementary Fig. 4c**), respectively. The temperature at which $\rho_{yx}(0)$ disappears is the Curie temperature (T_C) of the heterostructure. However, $\rho_{xx}(0)$ decreases and then increases with increasing temperature. The “upturn” feature at low temperatures is possibly induced by the Kondo effect in the magnetically doped TI samples [3].

Supplementary Note 3. More transport results of Sb_2Te_3/V-doped Sb_2Te_3 heterostructures

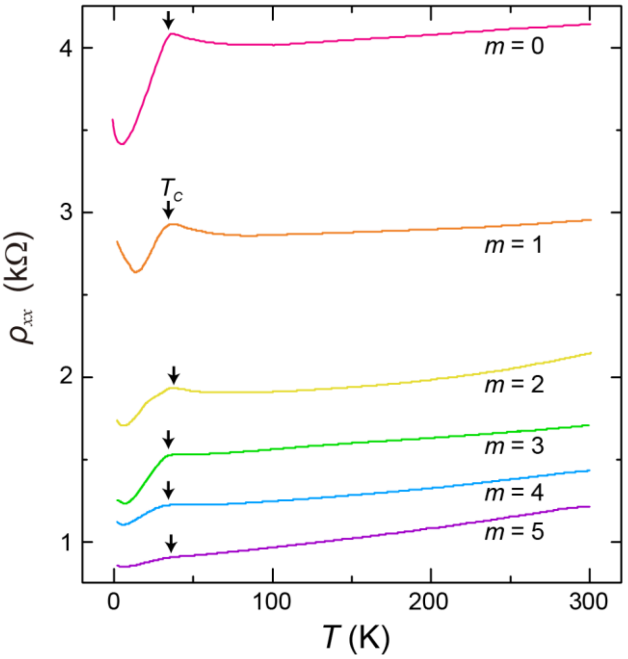

Supplementary Fig. 5| Temperature dependence of ρ_{xx} of the *m* QL Sb_2Te_3/5 QL $Sb_{1.9}V_{0.1}Te_3$ bilayer samples. The arrows indicates the Curie temperature T_C of these samples.

Supplementary Fig. 5 shows the T dependence of ρ_{xx} of the m QL Sb_2Te_3/5 QL $Sb_{1.9}V_{0.1}Te_3$ (m = 0, 1, 2, 3, 4, and 5) bilayer films. All these curves show similar features. The ρ_{xx} initially decreases with lowing T, indicating its metallic behavior. An upturn is observed in the low T region, which is likely induced by the Kondo effect in the magnetically doped TI samples[3]. Moreover, the hump features observed at T ~ 40 K is a result of spin-disorder scattering and reveal the Curie temperature (T_C) of these samples[4, 5].

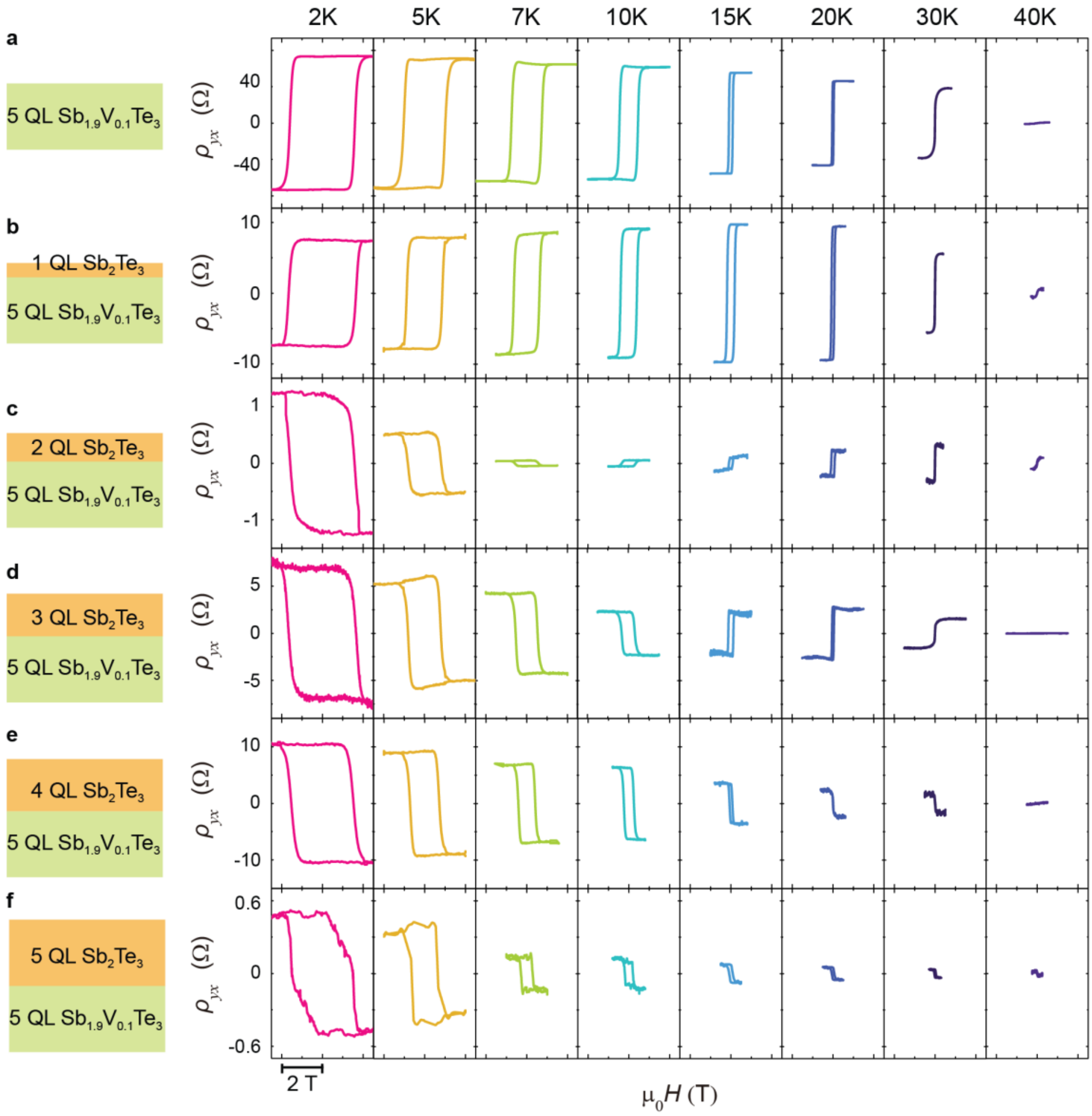

Supplementary Fig. 6| Hall traces of *m* QL Sb_2Te_3/5 QL $Sb_{1.9}V_{0.1}Te_3$ heterostructures at different temperatures. (a) $m = 0$, (b) $m = 1$, (c) $m = 2$, (d) $m = 3$, (e) $m = 4$, and (f) $m = 5$.

Supplementary Fig. 6 displays the $\mu_0 H$ dependence of ρ_{yx} of the *m* QL Sb_2Te_3/5 QL $Sb_{1.9}V_{0.1}Te_3$ heterostructures at different temperatures. For the $m = 0$ and $m = 1$ samples, the sign of the AH hysteresis loops is positive (i.e. $\rho_{yx} > 0$ for $M > 0$) throughout the ferromagnetic phase. For the $m = 0$ sample, $\rho_{yx}(0)$ decreases monotonically with increasing temperature, while the $\rho_{yx}(0)$ of the $m = 1$ sample first increases and then decreases with increasing temperature showing a maximum at T =15 K. For the $m = 2$ and $m = 3$ samples, the sign of the AH hysteresis loops is negative at low temperatures and becomes positive at high temperatures. With increasing temperature, the magnitude of $\rho_{yx}(0)$ decreases first and increases and then decreases. For the $m = 4$ and $m = 5$ samples, the sign of the AH hysteresis

loops is negative (i.e. $\rho_{yx} < 0$ for $M > 0$) in the entire ferromagnetic phase. The absolute value of $\rho_{yx}(0)$) decreases monotonically with increasing temperature. The temperature dependence of $\rho_{yx}(0)$ of all m QL Sb_2Te_3/5 QL $Sb_{1.9}V_{0.1}Te_3$ heterostructures (with m=0, 1, 2, 3, 4, and 5) are summarized in **Fig. 2f** of the main text.

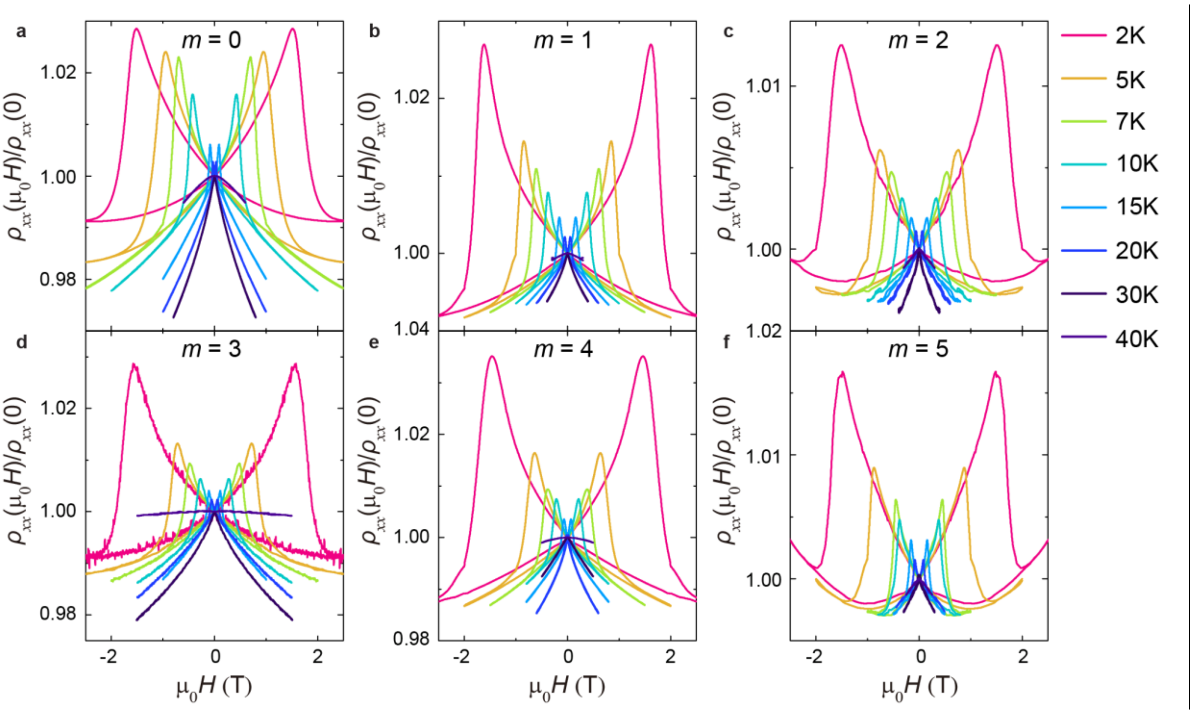

Supplementary Fig. 7| Normalized magnetoresistance $\rho_{xx}(\mu_0 H)/\rho_{xx}(0)$ of the *m* QL Sb_2Te_3/5 QL $Sb_{1.9}V_{0.1}Te_3$ heterostructures at different temperatures. (a) m = 0, (b) m = 1, (c) m = 2, (d) m = 3, (e) m = 4, and (f) m = 5.

Supplementary Fig. 7 shows the $\mu_0 H$ dependence of normalized magnetoresistance $\rho_{xx}(H)/\rho_{xx}(0)$ of the m QL Sb_2Te_3/5 QL $Sb_{1.9}V_{0.1}Te_3$ heterostructures at different temperatures. At T =2 K, all MR curves display the butterfly features, implying the formation of long-range ferromagnetic orders in all these samples. The disappearance of the butterfly structures at T = 40 K indicates the T_C of all these samples is ~ 40 K.

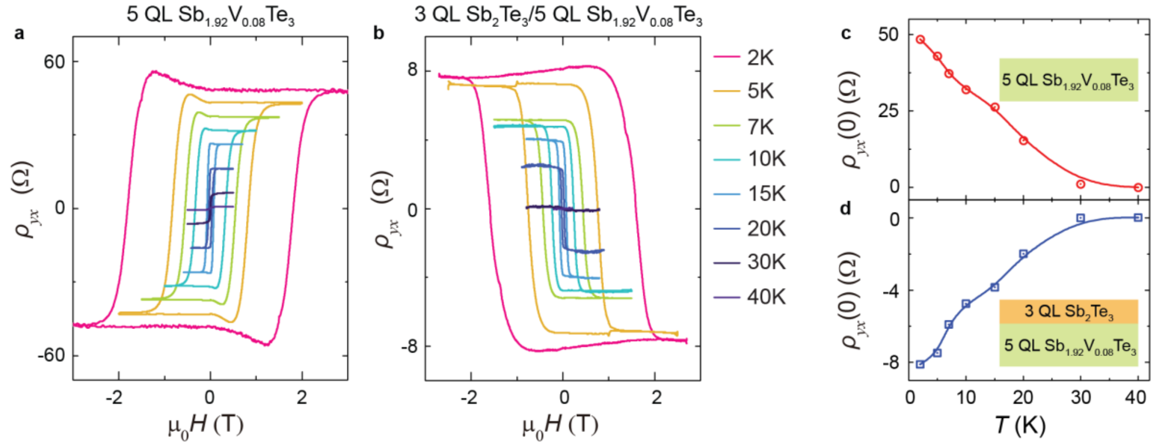

Supplementary Fig. 8| Hall traces of 5 QL $Sb_{1.92}V_{0.08}Te_3$ and 3 QL Sb_2Te_3/5 QL $Sb_{1.92}V_{0.08}Te_3$ samples at different temperatures. (a) 5 QL $Sb_{1.92}V_{0.08}Te_3$ film. (b) 3 QL Sb_2Te_3/5 QL $Sb_{1.92}V_{0.08}Te_3$ heterostructure. (c) Temperature dependence of $\rho_{yx}(0)$ of the 5 QL $Sb_{1.92}V_{0.08}Te_3$ film. (d) Temperature dependent $\rho_{yx}(0)$ of the 3 QL Sb_2Te_3/5 QL $Sb_{1.92}V_{0.08}Te_3$ heterostructure. The hump feature observed in the $Sb_{1.92}V_{0.08}Te_3$ film around the coercive field μ_0H_c is probably a result of the formation of the chiral spin textures in this sample.

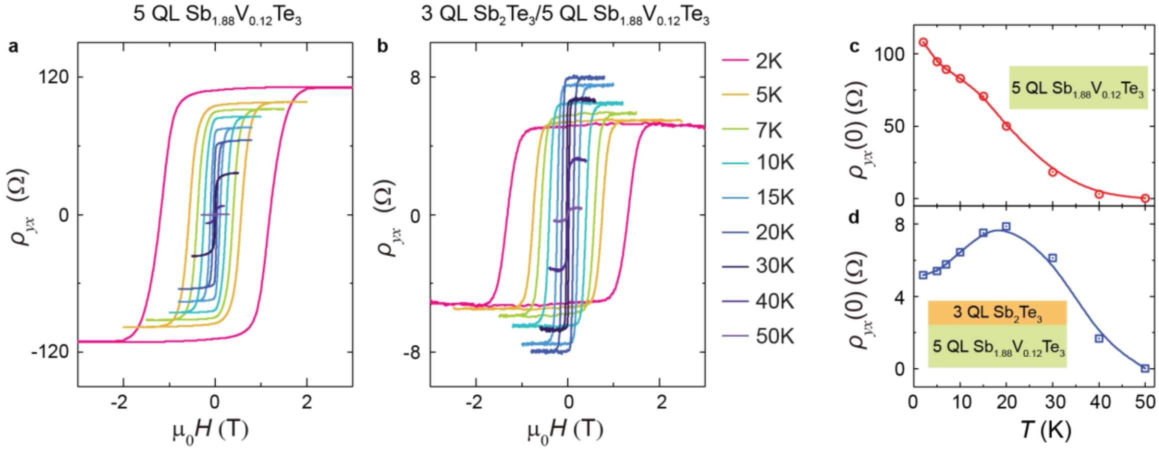

Supplementary Fig. 9| Hall traces of 5 QL $Sb_{1.88}V_{0.12}Te_3$ and 3 QL Sb_2Te_3/5 QL $Sb_{1.88}V_{0.12}Te_3$ samples at different temperatures (a) 5 QL $Sb_{1.88}V_{0.12}Te_3$ film. (b) 3 QL Sb_2Te_3/5 QL $Sb_{1.88}V_{0.12}Te_3$ heterostructure. (c) Temperature dependence of $\rho_{yx}(0)$ of the 5 QL $Sb_{1.88}V_{0.12}Te_3$ film. (d) Temperature dependence of $\rho_{yx}(0)$ of the 3 QL Sb_2Te_3/5 QL $Sb_{1.88}V_{0.12}Te_3$ heterostructure.

Supplementary Fig. 8a displays μ_0H dependence of ρ_{yx} of a 5 QL $Sb_{1.92}V_{0.08}Te_3$ film. The AH sign of the $Sb_{1.92}V_{0.08}Te_3$ sample is positive in the ferromagnetic phase. However, the

AH sign of the 3 QL Sb_2Te_3/5 QL $Sb_{1.92}V_{0.08}Te_3$ heterostructure is negative (**Supplementary Fig. 8b**). The absolute value of $\rho_{yx}(0)$ in both $Sb_{1.92}V_{0.08}Te_3$ and $Sb_2Te_3/Sb_{1.92}V_{0.08}Te_3$ samples decreases monotonically, as shown in **Supplementary Figs. 8c** and **8d**. Both samples share the same T_C ~ 30K, where $\rho_{yx}(0)$ vanishes.

Supplementary Figs. 9a and 9b show the Hall traces of a 5 QL $Sb_{1.88}V_{0.12}Te_3$ film and a 3 QL Sb_2Te_3/5 QL $Sb_{1.88}V_{0.12}Te_3$ heterostructure. Both samples show a positive AH sign in the ferromagnetic phase. $\rho_{yx}(0)$ of the 5 QL $Sb_{1.88}V_{0.12}Te_3$ film decreases monotonically with increasing temperature (**Supplementary Fig. 9c**), while $\rho_{yx}(0)$ of the 3 QL Sb_2Te_3/5 QL $Sb_{1.88}V_{0.12}Te_3$ heterostructure first increases and then decreases with increasing temperature (**Supplementary Fig. 9d**). Both samples share the same T_C ~ 50K.

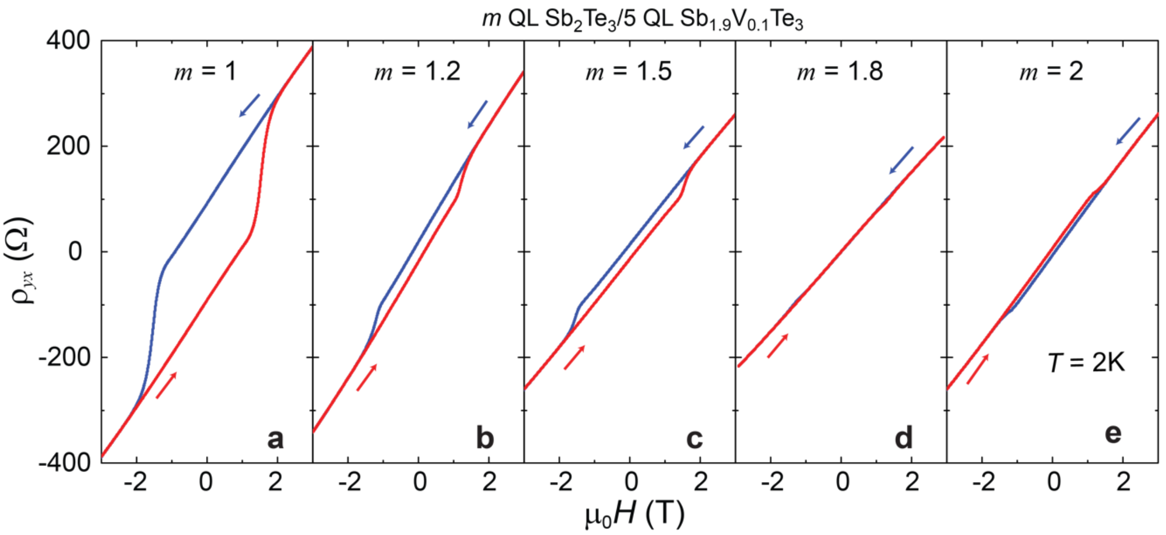

Supplementary Fig. 10 | Hall traces of *m* QL Sb_2Te_3/5 QL $Sb_{1.9}V_{0.1}Te_3$ bilayer heterostructures. (a-e) μ_0H dependence of ρ_{yx} in *m* QL Sb_2Te_3/5 QL $Sb_{1.9}V_{0.1}Te_3$ bilayer heterostructures. *m* = 1 (a); *m* = 1.2 (b); *m* =1.5 (c); *m* =1.8 (d); *m* =2 (e). All measurements were taken at *T* = 2 K. The Hall trace become linear at *m* = 1.8. The arrows indicate the magnetic field sweep directions.

We systematically synthesized a series of samples with different thicknesses of the undoped TI layer from 1 to 2 quintuple layers (QLs) and found that the critical thickness for the zero AH resistance ρ_{yx} at V_g= 0 V is ~1.8 QL (**Supplementary Fig. 10**).

Supplementary Note 4. Gate-tuned magnetotransport results of Sb_2Te_3/V-doped Sb_2Te_3 heterostructures

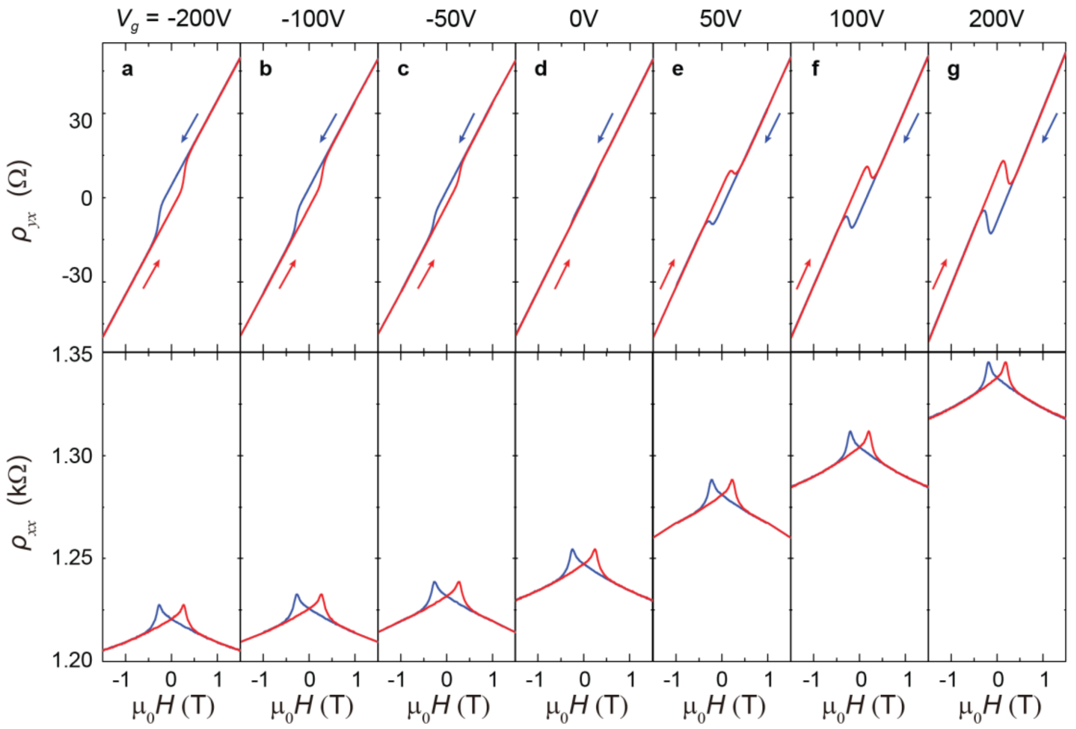

Supplementary Fig. 11| $\mu_0 H$ dependence of ρ_{yx} and ρ_{xx} of 3 QL Sb_2Te_3/5 QL $Sb_{1.9}V_{0.1}Te_3$ heterostructure under different gates measured at T = 11 K. (a) V_g =-200V. (b) V_g =-100V. (c) V_g =-50V. (d) V_g =0V. (e) V_g =+50V. (f) V_g =+100V. (g) V_g = +200V. The arrows indicate the magnetic field sweep directions.

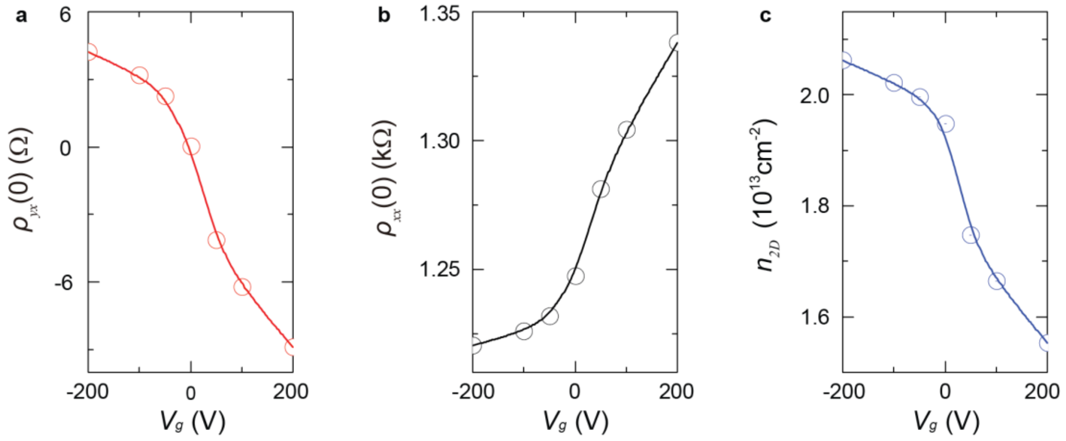

Supplementary Fig. 12| V_g dependence of $\rho_{yx}(0)$, $\rho_{xx}(0)$, and carrier density n of the 3 QL Sb_2Te_3/5 QL $Sb_{1.9}V_{0.1}Te_3$ heterostructure. (a) $\rho_{yx}(0)$. (b) $\rho_{xx}(0)$. (c) Carrier density n_{2D}.

Supplementary Fig. 11 shows $\mu_0 H$ dependence of ρ_{yx} of the 3 QL Sb_2Te_3/5 QL $Sb_{1.9}V_{0.1}Te_3$ heterostructure without subtracting the ordinary Hall component under different V_gs measured at T = 11 K. We find that positive V_g favors negative AH effect and the negative gate voltage V_g favors positive AH effect. The tuning of V_g from -200V to +200V reduces the AH resistance at zero magnetic field $\rho_{yx}(0)$ (**Supplementary Fig. 12a**) and enhances the longitudinal resistance at zero magnetic field $\rho_{xx}(0)$ (**Supplementary Fig. 12b**). The calculated carrier densities n_{2D} of the sample is found to decrease when V_g is tuned from -200V to +200V(**Supplementary Fig. 12c**). All these behaviors indicate that the chemical potential of this sample is crossing the bulk valence bands upon the tuning of V_g.

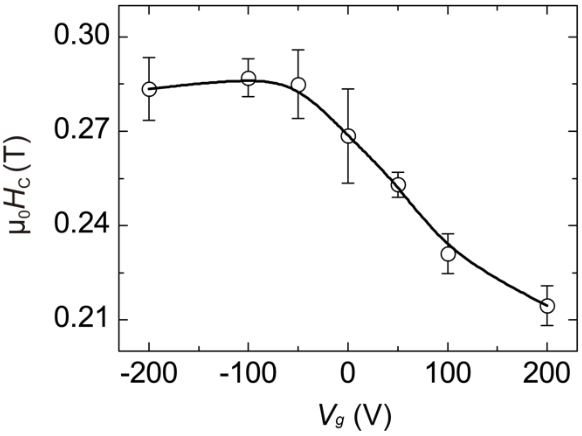

Supplementary Fig. 13 | V_g dependence of the coercive field ($\mu_0 H_c$) of the 3 QL Sb_2Te_3/5 QL $Sb_{1.9}V_{0.1}Te_3$ bilayer samples. All measurements were taken at T =11 K.

Supplementary Fig. 13 shows V_g dependence of the coercive field ($\mu_0 H_c$) obtained from **Supplementary Fig. 11**. With decreasing V_g from +200 V to -200 V, $\mu_0 H_c$ initially increases and then becomes a constant, indicating that the ferromagnetic property of the 3 QL Sb_2Te_3/5 QL $Sb_{1.9}V_{0.1}Te_3$ bilayer film is carrier-dependent.

Supplementary Note 5. Stacking sequence dependence of transport properties

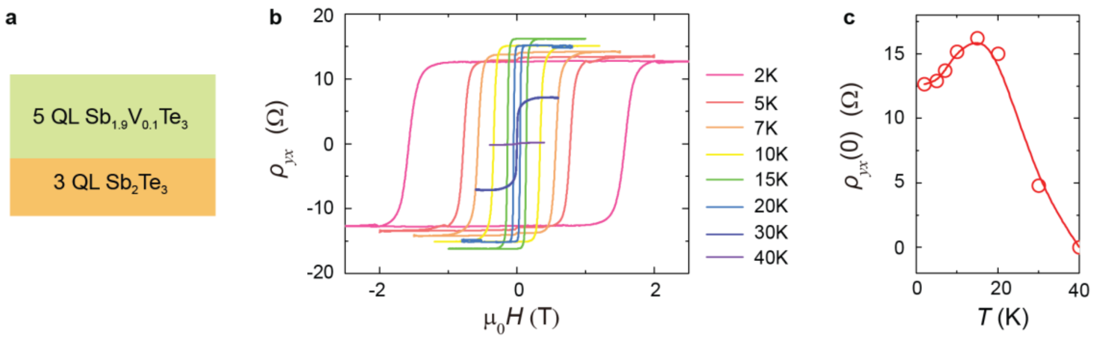

Supplementary Fig. 14| Hall traces of 5 QL $Sb_{1.9}V_{0.1}Te_3$/3 QL Sb_2Te_3 heterostructure at different temperatures. (a) Schematic for the 5 QL $Sb_{1.9}V_{0.1}Te_3$/3 QL Sb_2Te_3 heterostructure. (b) Hall traces of the 5 QL $Sb_{1.9}V_{0.1}Te_3$/3 QL Sb_2Te_3 heterostructure at different temperatures. (c) Temperature dependence of $\rho_{yx}(0)$.

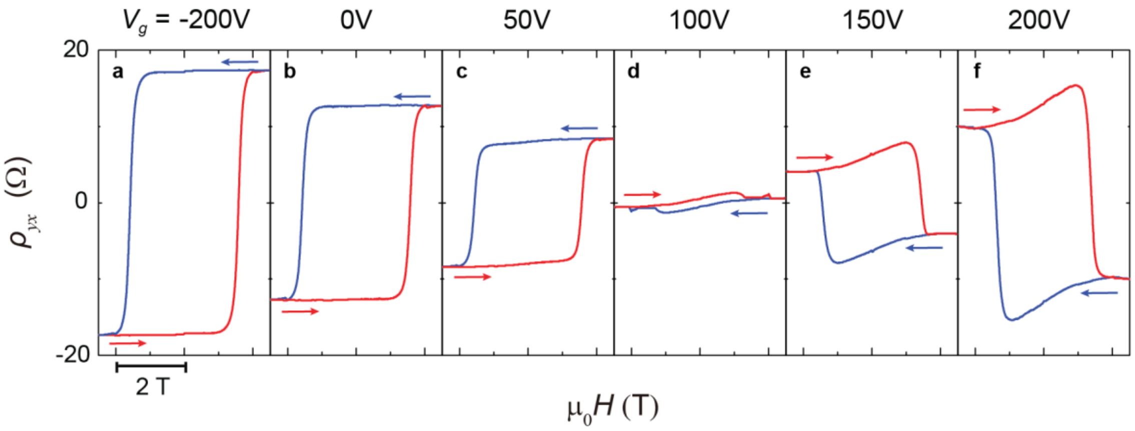

Supplementary Fig. 15| Hall traces of 5 QL $Sb_{1.9}V_{0.1}Te_3$/3 QL Sb_2Te_3 heterostructure measured at different V_g. (a) V_g =-200 V. (b) V_g =0 V. (c) V_g =+50 V. (d) V_g = +100 V. (e) V_g =+150 V. (f) V_g =+200 V. (g) V_g = +200 V. The arrows indicate the magnetic field sweep directions.

We also carried out magnetotransport measurements on the 5 QL $Sb_{1.9}V_{0.1}Te_3$/3 QL Sb_2Te_3 heterostructure sample (**Supplementary Fig. 14a**). Unlike the negative AH sign in the 3 QL Sb_2Te_3/5 QL $Sb_{1.9}V_{0.1}Te_3$ sample (**Supplementary Fig. 6d**), the AH sign of the 5 QL $Sb_{1.9}V_{0.1}Te_3$/3 QL Sb_2Te_3 sample is positive in the entire temperature range of the experiment. The magnitude of $\rho_{yx}(0)$ first increases and then decreases with increasing temperature, as

shown in **Supplementary Fig. 14c**. The opposite AH sign in these two samples with different stacking sequences is probably due to the $SrTiO_3$ substrates induced band bending effect, which may shift the chemical potential of the 5 QL $Sb_{1.9}V_{0.1}Te_3$ layer. We next investigated the V_g dependence of the Hall trace of the 5 QL $Sb_{1.9}V_{0.1}Te_3$/3 QL Sb_2Te_3 heterostructure at T = 2 K (**Supplementary Fig. 15**). We find that when V_g is tuned from -200V to +200V, the AH sign changes from being positive to negative and the magnitude of $\rho_{yx}(0)$ shows a minimum at V_g =+100V (**Supplementary Fig. 15d**).

Supplementary Note 6. More transport results of Cr-doped Sb_2Te_3/Sb_2Te_3/V-doped Sb_2Te_3 heterostructures

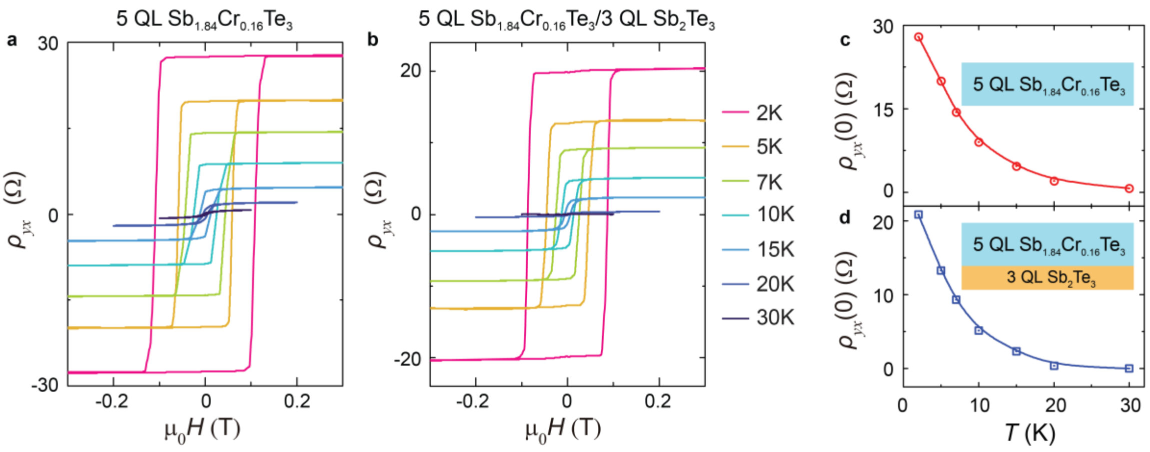

Supplementary Fig. 16| Hall traces of 5 QL $Sb_{1.84}Cr_{0.16}Te_3$ and 5 QL $Sb_{1.84}Cr_{0.16}Te_3$/3 QL Sb_2Te_3 samples at different temperatures. (a) 5 QL $Sb_{1.84}Cr_{0.16}Te_3$ film. (b) 5 QL $Sb_{1.84}Cr_{0.16}Te_3$/3 QL Sb_2Te_3 heterostructure. (c) Temperature-dependent $\rho_{yx}(0)$ of the 5 QL $Sb_{1.84}Cr_{0.16}Te_3$ film. (d) Temperature-dependent $\rho_{yx}(0)$ of the 5 QL $Sb_{1.84}Cr_{0.16}Te_3$/3 QL Sb_2Te_3 heterostructure.

As noted in the main text, to realize the artificial topological Hall (TH) effect in the sandwich samples, we need two decoupled ferromagnetic orders with opposite AH signs and different $\mu_0 H_c$. Since the AH effect with negative sign can appear in the Sb_2Te_3/V-doped Sb_2Te_3 bilayer heterostructure. Here we studied the AH effects of both Cr-doped TI film and Cr-doped TI/TI heterostructure. **Supplementary Figs. 16a** and **16b** show $\mu_0 H$ dependence of ρ_{yx} of the 5 QL $Sb_{1.84}Cr_{0.16}Te_3$ and 5 QL $Sb_{1.84}Cr_{0.16}Te_3$/3QL Sb_2Te_3 samples at different temperatures, respectively. At the base temperature T = 2 K, both samples show a "square-like" AH hysteresis loop with the positive sign, demonstrating the formation of the long-range FM order in both samples. $\rho_{yx}(0)$ decreases with increasing temperature and vanishes at T_C ~ 30 K (**Supplementary Fig. 16c** and **16d**). Moreover, $\mu_0 H_c$ of the $Sb_{1.84}Cr_{0.16}Te_3$/Sb_2Te_3 layer is ~ 0.09 T, much smaller than $\mu_0 H_c$~1.6 T of the Sb_2Te_3/$Sb_{1.92}V_{0.08}Te_3$ bilayer film (**Supplementary Fig. 8b**). Therefore, we observed an artificial "TH effect" in the Hall traces of the 5 QL $Sb_{1.84}Cr_{0.16}Te_3$/3 QL Sb_2Te_3/5 QL $Sb_{1.92}V_{0.08}Te_3$ sandwich heterostructure (Fig. 5 of the main text).

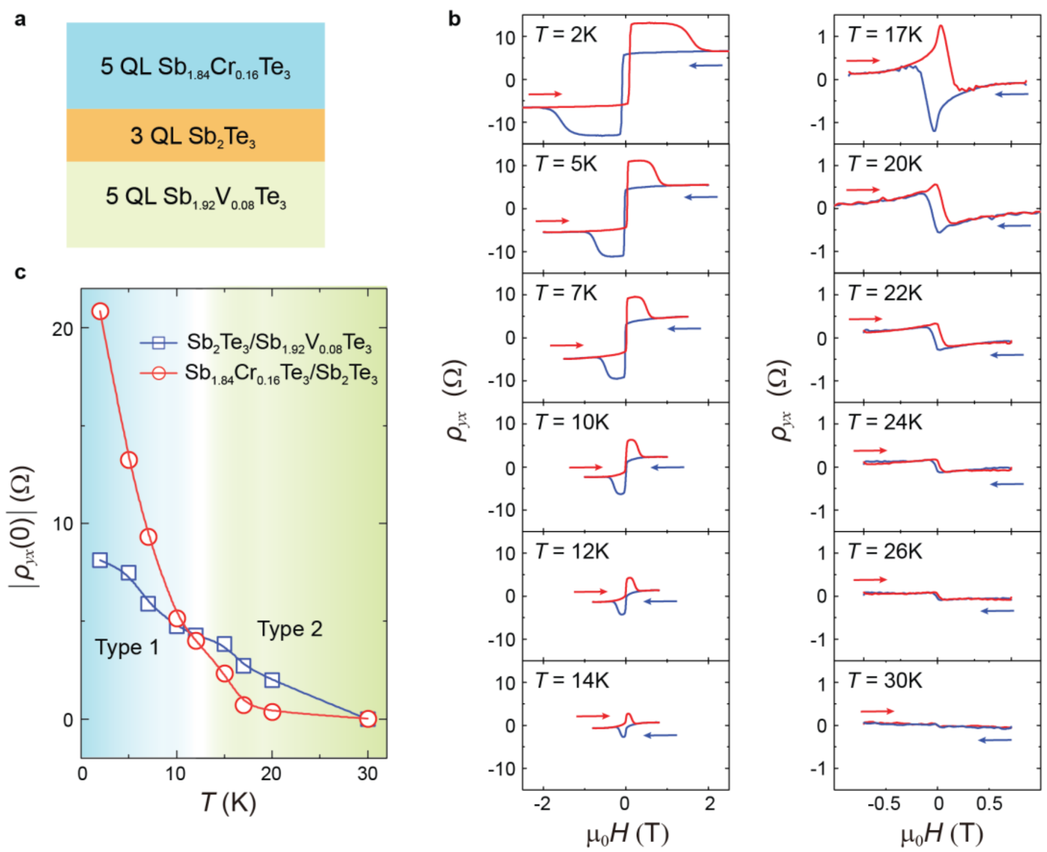

Supplementary Fig. 17| The artificial "topological Hall effect" observed in the 5 QL $Sb_{1.84}Cr_{0.16}Te_3$/3 QL Sb_2Te_3/5 QL $Sb_{1.92}V_{0.08}Te_3$ sandwich heterostructure. (a) Schematic for the 5 QL $Sb_{1.84}Cr_{0.16}Te_3$/3 QL Sb_2Te_3/5 QL $Sb_{1.92}V_{0.08}Te_3$ sandwich sample. (b) $\mu_0 H$ dependence of ρ_{yx} of the 5 QL $Sb_{1.84}Cr_{0.16}Te_3$/3 QL Sb_2Te_3/5 QL $Sb_{1.92}V_{0.08}Te_3$ heterostructure measured at different temperatures. (c) The temperature depdence of the absolute value of ρ_{yx} (0) (i.e. $|\rho_{yx}$ (0)$|$) of the 5 QL $Sb_{1.84}Cr_{0.16}Te_3$/3 QL Sb_2Te_3 and 3 QL Sb_2Te_3/5 QL $Sb_{1.92}V_{0.08}Te_3$ samples. The arrows in (b) indicate the magnetic field sweep directions.

Supplementary Fig. 17 shows the temperature evolution of the artificial "TH effect" in the 5 QL $Sb_{1.84}Cr_{0.16}Te_3$/3 QL Sb_2Te_3/5 QL $Sb_{1.92}V_{0.08}Te_3$ heterostructure. With increasing temperature, the TH-like feature changes from Type 1 to Type 2 at $T\sim$ 12 K and becomes unobservable for $T >$ 22 K (**Supplementary Fig. 17b**). The definitions of Type 1 and Type 2 TH like features can be found in the main text. **Supplementary Fig. 17c** shows the temperature dependence of the absolute value of ρ_{yx} (0) (i.e. $|\rho_{yx}$ (0)$|$) of the 5 QL $Sb_{1.84}Cr_{0.16}Te_3$/3 QL Sb_2Te_3 and 3 QL Sb_2Te_3/5 QL $Sb_{1.92}V_{0.08}Te_3$ samples. For $T <$ 12 K, $|\rho_{yx}$ (0)$|$ of 5 QL $Sb_{1.84}Cr_{0.16}Te_3$/3 QL Sb_2Te_3 is larger than $|\rho_{yx}$ (0)$|$ of 3 QL Sb_2Te_3/5 QL

$Sb_{1.92}V_{0.08}Te_3$ sample, so we observe "Type 1" TH like loop (**Supplementary Fig. 17b**). For $T \geq 12$ K, $|\rho_{yx}(0)|$ of 5 QL $Sb_{1.84}Cr_{0.16}Te_3$/3 QL Sb_2Te_3 is larger than $|\rho_{yx}(0)|$ of 3 QL Sb_2Te_3/5 QL $Sb_{1.92}V_{0.08}Te_3$ sample, so "Type 2" TH like feature is observed (see **Figs. 5a** and **5b** of the main text). Our results demonstrate that the TH "hump" feature in our Cr-doped Sb_2Te_3/Sb_2Te_3/V-doped Sb_2Te_3 sandwich heterostructures originates from the superposition of two AH loops with opposite signs rather than from the formation of chiral spin textures in our samples.

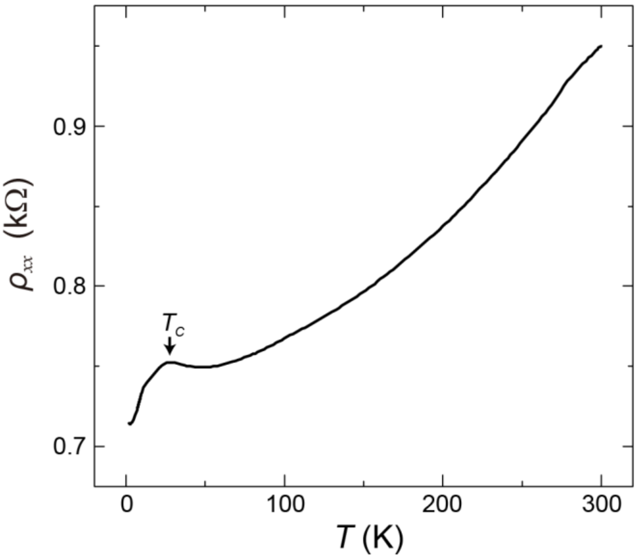

Supplementary Fig. 18| Temperature dependence of ρ_{xx} of the 5 QL $Sb_{1.84}Cr_{0.16}Te_3$/3 QL Sb_2Te_3/5 QL $Sb_{1.92}V_{0.08}Te_3$ sandwich sample.

Supplementary Fig. 18 shows the T dependence of ρ_{xx} of the 5 QL $Sb_{1.84}Cr_{0.16}Te_3$/3 QL Sb_2Te_3/5 QL $Sb_{1.92}V_{0.08}Te_3$ sandwich sample. ρ_{xx} shows a metallic behavior with a hump feature at $T \sim 30$K.

Supplementary Note 7. Transport results of the 5 QL $Sb_{1.84}Cr_{0.16}Te_3$/5 QL $Sb_{1.92}V_{0.08}Te_3$ heterostructure

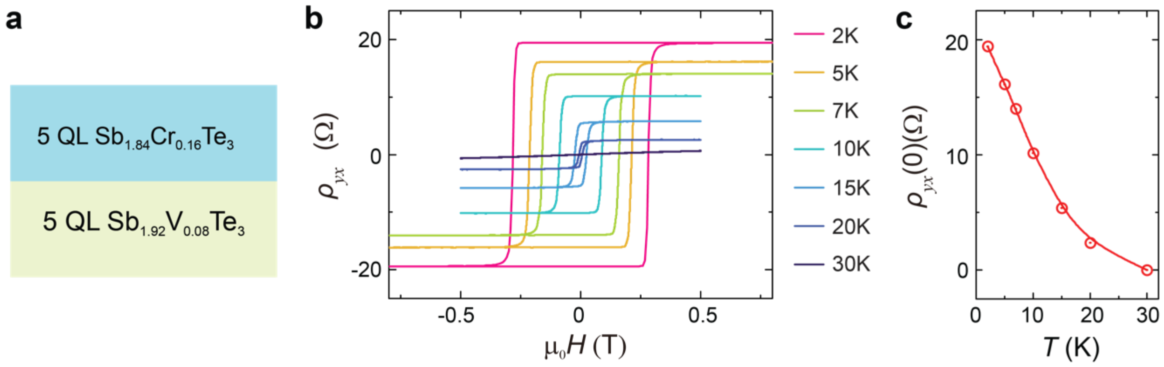

Supplementary Fig. 19| Hall traces of 5 QL $Sb_{1.84}Cr_{0.16}Te_3$/5 QL $Sb_{1.92}V_{0.08}Te_3$ bilayer. (a) Schematic for the 5 QL $Sb_{1.84}Cr_{0.16}Te_3$/5 QL $Sb_{1.92}V_{0.08}Te_3$ bilayer sample. (b) Hall traces of the 5 QL $Sb_{1.84}Cr_{0.16}Te_3$/5 QL $Sb_{1.92}V_{0.08}Te_3$ bilayer sample measured at different temperatures. (c) Temperature dependence of $\rho_{yx}(0)$.

We also carried out Hall measurements on the 5 QL $Sb_{1.84}Cr_{0.16}Te_3$/5 QL $Sb_{1.92}V_{0.08}Te_3$ bilayer sample (**Supplementary Fig. 19a**). As note in the main text, the middle 3 QL Sb_2Te_3 layer weakens the interlayer exchange coupling between the top and the bottom magnetic layers in the 5 QL $Sb_{1.84}Cr_{0.16}Te_3$/3 QL Sb_2Te_3/5 QL $Sb_{1.92}V_{0.08}Te_3$ sandwich heterostructure. Therefore, the absence of the middle 3QL Sb_2Te_3 layer (i.e. the Cr-doped TI/V-doped TI bilayer sample) will make the top $Sb_{1.84}Cr_{0.16}Te_3$ layer and the bottom $Sb_{1.92}V_{0.08}Te_3$ layer couple directly and show a single hysteresis loop, as shown in **Supplementary Fig. 19b**. $\rho_{yx}(0)$ decreases with increasing temperature and vanishes at $T_C \sim 30$ K (**Supplementary Fig. 19c**).

Supplementary Note 8. Electronic structures in V- and Cr-doped Sb_2Te_3 with different dopant positions

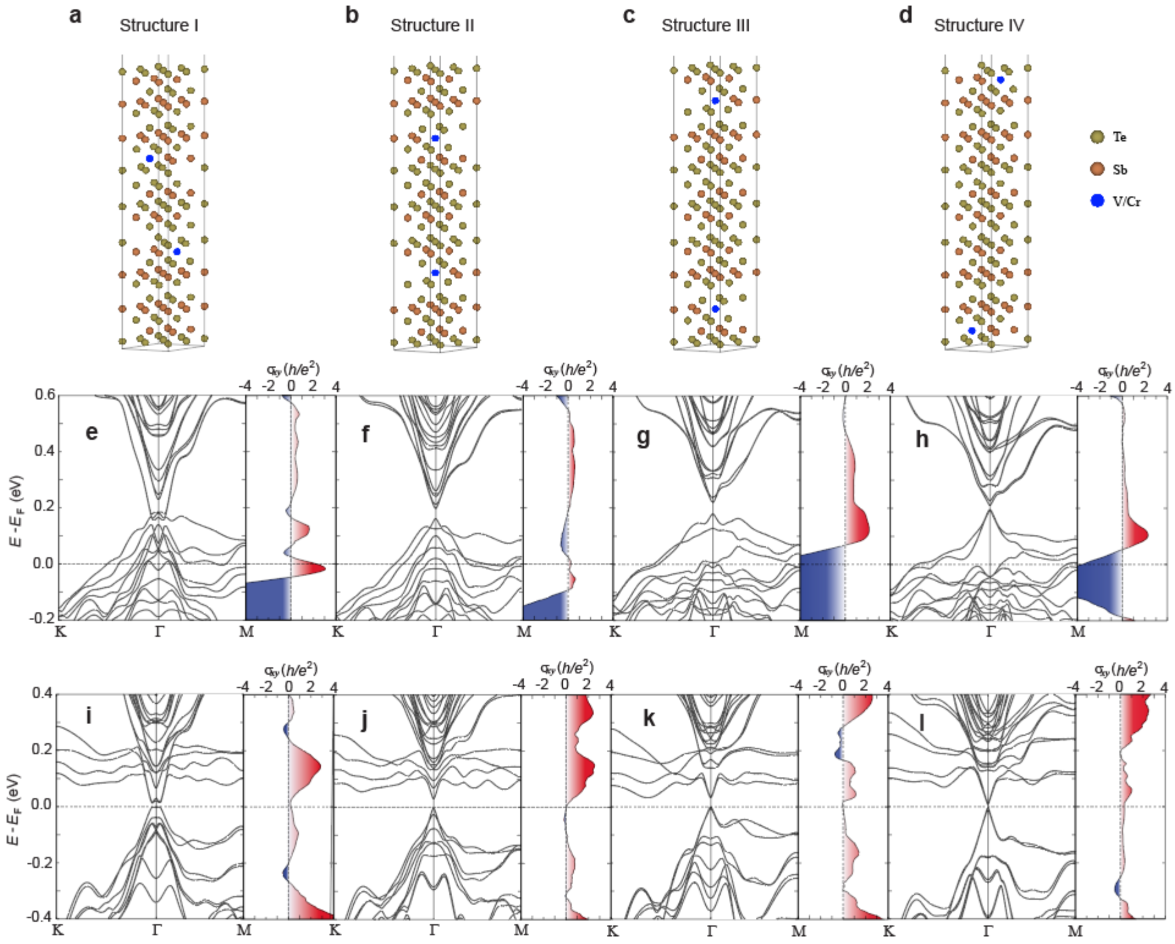

Supplementary Fig. 20| Band structures and AH conductance of 5QL $Sb_{1.9}V_{0.1}Te_3$ and 5QL $Sb_{1.9}Cr_{0.1}Te_3$ for four different dopant positions. (a, b, c, d) Schematics for four substitutional positions of two V/Cr atoms in a 2×2×5 supercell, labeled as Structure I (a), Structure II (b), Structure III (c), Structure IV (d). (e, f, g, h) The calculated band structures (left) and corresponding AH conductance σ_{xy} (right) of 5QL $Sb_{1.9}V_{0.1}Te_3$ film with Structure I (e), Structure II (f), Structure III (g), Structure IV (h). (i, j, k, l) The calculated band structures (left) and corresponding AH conductance σ_{xy} (right) of 5QL $Sb_{1.9}Cr_{0.1}Te_3$ film with Structure I (i), Structure II (j), Structure III (k), Structure IV (l).

In our prior studies, we have demonstrated that Cr and V dopants are distributed uniformly in the V- and Cr-doped Sb_2Te_3 films[1, 6, 7]. However, when we investigate the electronic structures of 5 QL V- and Cr-doped Sb_2Te_3 films through density functional theory

(DFT), we need to make a supercell of 5 QL Sb_2Te_3 with the V or Cr dopants. We considered here four substitutional positions of two V/Cr atoms in a 2×2×5 supercell and labeled them as Structure I (**Supplementary Fig. 20a**), Structure II (**Supplementary Fig. 20b**), Structure III (**Supplementary Fig. 20c**), Structure IV (**Supplementary Fig. 20d**). For the 5QL V-doped Sb_2Te_3 films, the relative formation energies are 0 eV for Structure I, -0.01 eV for Structure II, 0.03 eV for Structure III and 0.30 eV for Structure IV, respectively. Therefore, Structure II is the optimal substitutional position for 5QL V-doped Sb_2Te_3 films. We then calculated the band structures and the AH conductance σ_{xy} of 5QL $Sb_{1.9}V_{0.1}Te_3$ with these four substitutional structures, as shown in **Supplementary Figs. 20e** to **20h**. From our theoretical calculations, we found that there is no QAH effect regime for structure II. A trivial gap is formed between the bulk conduction and valence bands, where the Hall conductance σ_{xy} is zero. We speculate this gap is induced by the hybridization between top and bottom surface states. For Structure I (i.e. two V atoms located at the fourth outermost Sb layers), since the V atoms are far away from the top and bottom surfaces, both show the gapless surface states. In contrast, Structure III (i.e. two V atoms located at the second outermost Sb layers) and Structure IV (i.e. two V atoms located at the outermost Sb layers) both show the magnetic exchange gap and the QAH effect appears when the chemical potential is tuned into the magnetic exchange gap.

As a comparison, we also calculated the band structures and the AH conductance σ_{xy} of 5QL $Sb_{1.9}Cr_{0.1}Te_3$ with these four substitutional structures, as shown in **Supplementary Fig. 20i** to **20l**. For the 5QL Cr-doped Sb_2Te_3 films, the relative formation energies are 0 eV for Structure I, 0.02 eV for Structure II, 0.07 eV for Structure III and 0.18 eV for Structure IV, respectively. We can see that for 5QL Cr-doped Sb_2Te_3 films, Structure I is the optimal substitutional position. Moreover, our calculations show that for both V and Cr dopants the formation energies between Structure I and Structure II are only very slightly different, this suggests that Structure I and Structure II may coexist in real experiments.

Supplementary Note 9. More theoretical results of V- and Cr-doped Sb_2Te_3

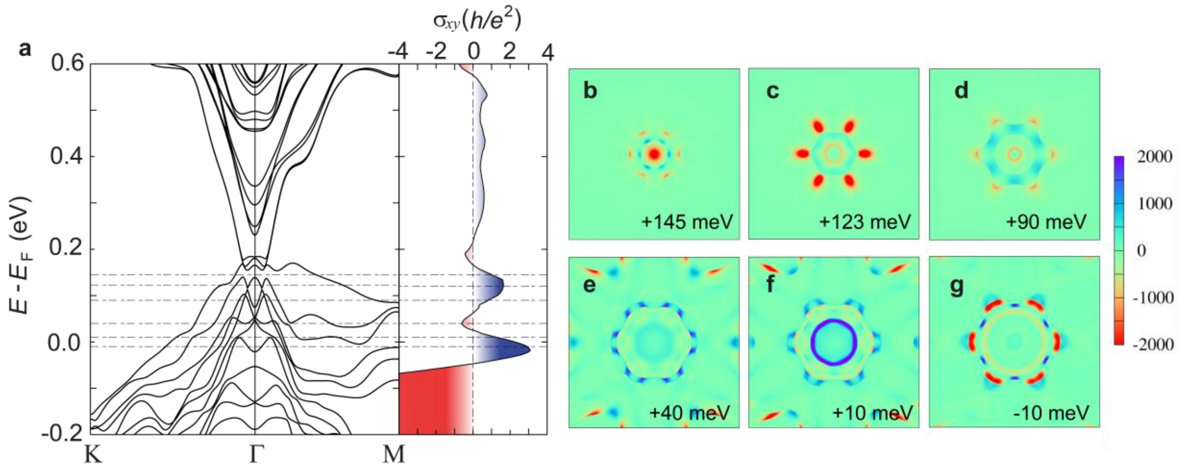

Supplementary Fig. 21| Band structure, AH conductance and Berry curvature of 5QL $Sb_{1.9}V_{0.1}Te_3$ with Structure I. (a) The calculated band structure (left) and corresponding Hall conductance σ_{xy} (right). (b, c, d, e, f, g) The calculated Berry curvature distribution of 5 QL $Sb_{1.9}V_{0.1}Te_3$ for chemical potential located at +145 meV(b), +123 meV(c), +90 meV(d), +40 meV(e), +10 meV(f), and -10 meV(g). The corresponding chemical potential positions are also labeled in (a) with dashed lines.

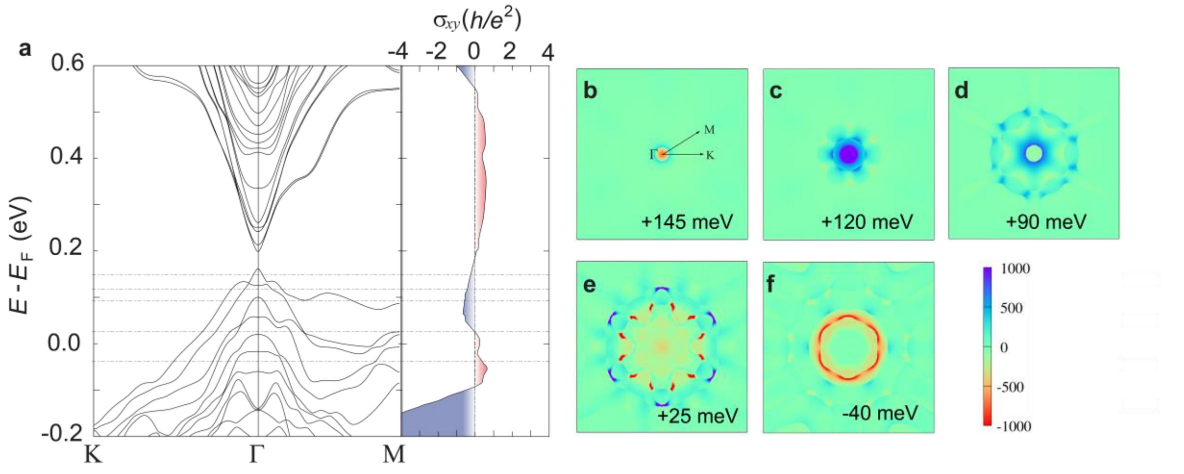

Supplementary Fig. 22| Band structure, AH conductance and Berry curvature of 5QL $Sb_{1.9}V_{0.1}Te_3$ with Structure I. (a) The calculated band structure (left) and corresponding Hall conductance σ_{xy} (right). (b, c, d, e, f) The calculated Berry curvature distribution of 5 QL $Sb_{1.9}V_{0.1}Te_3$ for chemical potential located at +145 meV(b), +120 meV(c), +90 meV(d), +25 meV(e), and -40 meV(f). The corresponding chemical potential positions are also labeled in (a) with dashed lines.

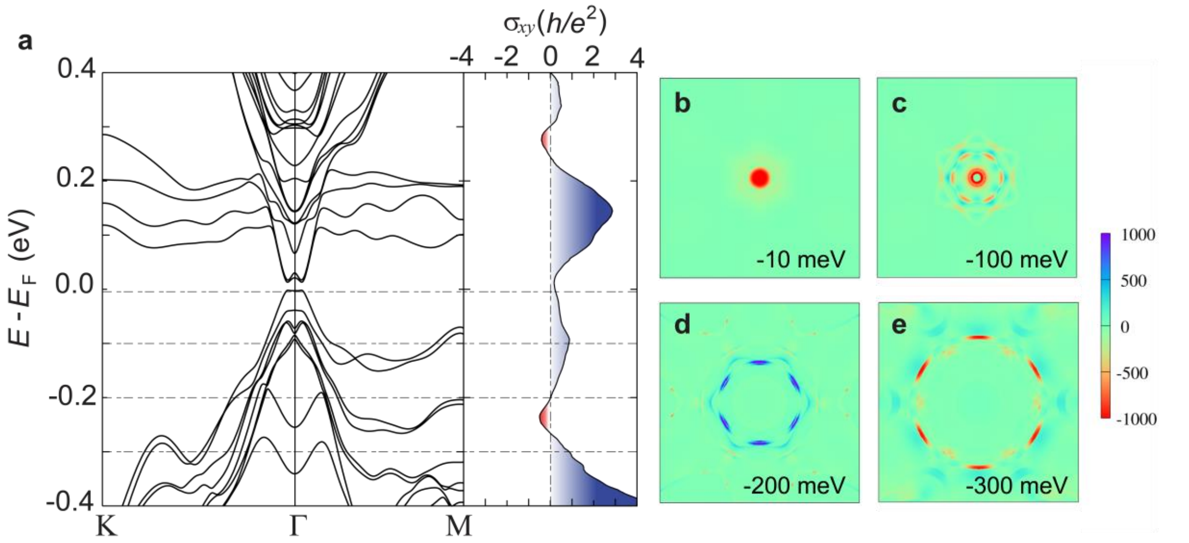

Supplementary Fig. 23| Band structure, AH conductance and Berry curvature of 5QL $Sb_{1.9}Cr_{0.1}Te_3$ with Structure I. (a) The calculated band structure (left) and corresponding Hall conductance σ_{xy} (right). (b, c, d, e) The calculated Berry curvature distribution of 5 QL $Sb_{1.9}Cr_{0.1}Te_3$ for chemical potential located at -10 meV(b), -100 meV(c), -200 meV(d), and -300 meV(e). The corresponding chemical potential positions are also labeled in (a) with dashed lines.

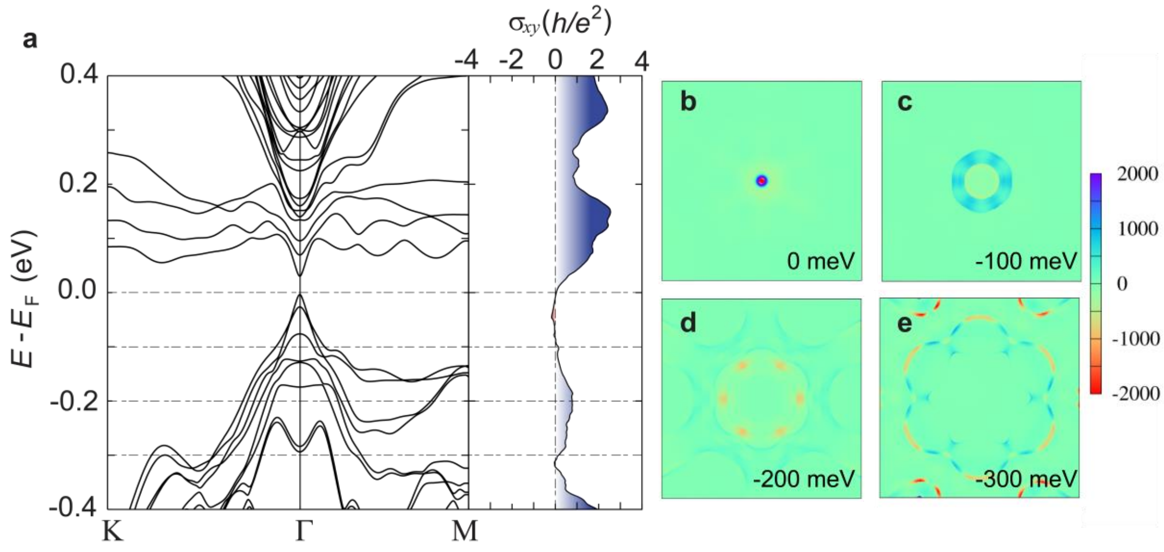

Supplementary Fig. 24| Band structure, AH conductance and Berry curvature of 5QL $Sb_{1.9}Cr_{0.1}Te_3$ with Structure II. (a) The calculated band structure (left) and corresponding Hall conductance σ_{xy} (right). (b, c, d, e) The calculated Berry curvature distribution of 5 QL $Sb_{1.9}Cr_{0.1}Te_3$ for chemical potential located at 0 meV(b), -100 meV(c), -200 meV(d), and -300 meV(e). The corresponding chemical potential positions are also labeled in (a) with

dashed lines.

Supplementary Figs. 21 and 22 shows the calculated band structure, the AH conductance σ_{xy}, and the Berry curvature of 5QL $Sb_{1.9}V_{0.1}Te_3$ with Structure I and Structure II, respectively. The sign of the AH conductance σ_{xy} can indeed be changed from being positive to negative by injecting electron carriers. We also theoretically studied why in the Cr-doped Sb_2Te_3, the sign of the AH conductance σ_{xy} is always positive upon tuning the chemical potential in our experiments. **Supplementary Figs. 23** and **24** show the calculated band structure, the AH conductance σ_{xy}, and the Berry curvature of 5QL $Sb_{1.9}Cr_{0.1}Te_3$ with Structure I and Structure II, respectively. We can see that the band structure of Cr-doped Sb_2Te_3 is quite different from that of V-doped Sb_2Te_3. For both configurations, the sign of the AH conductance σ_{xy} is always positive when the chemical potential is tuned from -200 meV to -100 meV. This energy range corresponds to the tunability by electric gating in real experiments[1, 6, 7]. Therefore, our calculations demonstrated that the sign of the AH conductance in V-doped Sb_2Te_3 can be changed from positive to negative, but in Cr-doped Sb_2Te_3 the AH sign is always positive within the range of chemical potential tunability, reflecting the difference of the band structures of the two systems.

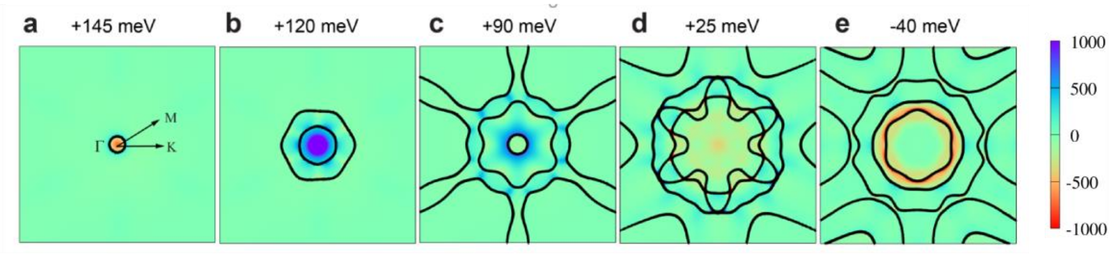

Supplementary Fig. 25| Berry curvature and Fermi surface of 5QL $Sb_{1.9}V_{0.1}Te_3$ with Structure II for various chemical potential levels. (a) +145 meV, (b) +120 meV, (c) +90 meV, (d) +25 meV, (e) -40 meV. The Fermi surfaces are shown in thick black lines and the background is the corresponding Berry curvature.

To link the Berry curvature with the detailed bands, we calculated the Fermi surface of the 5QL $Sb_{1.9}V_{0.1}Te_3$ with Structure II at the corresponding chemical potentials shown in **Supplementary Fig. 22**, as shown in **Supplementary Fig. 25**. **Supplementary Figs. 25d** and **25e** show that in the positive regions of the AH conductance σ_{xy} at E = +25 meV and E = -40 meV, where we can see that the positive Berry curvature peaks indeed correspond to the

band crossing or anti-crossing. Therefore, our calculations demonstrated that the sign change of the AH conductance σ_{xy} for 5QL $Sb_{1.9}V_{0.1}Te_3$ is a result of the accidental band crossing or anti-crossing in the valence bands, consistent with prior studies [8, 9].

Supplementary References

1. Chang C. Z., Zhao W. W., Kim D. Y., Zhang H. J., Assaf B. A., Heiman D., Zhang S. C., Liu C. X., Chan M. H. W., Moodera J. S. High-Precision Realization of Robust Quantum Anomalous Hall State in a Hard Ferromagnetic Topological Insulator. *Nat. Mater.* **14**, 473-477 (2015).

2. Xiao D., Jiang J., Shin J. H., Wang W. B., Wang F., Zhao Y. F., Liu C. X., Wu W. D., Chan M. H. W., Samarth N., Chang C. Z. Realization of the Axion Insulator State in Quantum Anomalous Hall Sandwich Heterostructures. *Phys. Rev. Lett.* **120**, 056801 (2018).

3. He H. T., Yang C. L., Ge W. K., Wang J. N., Dai X., Wang Y. Q. Resistivity minima and Kondo effect in ferromagnetic GaMnAs films. *Appl. Phys. Lett.* **87**, 162506 (2005).

4. Zhou Z. H., Chien Y. J., Uher C. Thin film dilute ferromagnetic semiconductors $Sb_{2-x}Cr_xTe_3$ with a Curie temperature up to 190 K. *Phys. Rev. B* **74**, 224418 (2006).

5. Wang F., Xiao D., Yuan W., Jiang J., Zhao Y.-F., Zhang L., Yao Y., Liu W., Zhang Z., Liu C., Shi J., Han W., Chan M. H. W., Samarth N., Chang C.-Z. Observation of Interfacial Antiferromagnetic Coupling between Magnetic Topological Insulator and Antiferromagnetic Insulator. *Nano Lett.* **19**, 2945-2952 (2019).

6. Chang C. Z., Zhang J. S., Liu M. H., Zhang Z. C., Feng X., Li K., Wang L. L., Chen X., Dai X., Fang Z., Qi X. L., Zhang S. C., Wang Y. Y., He K., Ma X. C., Xue Q. K. Thin Films of Magnetically Doped Topological Insulator with Carrier-Independent Long-Range Ferromagnetic Order. *Adv. Mater.* **25**, 1065-1070 (2013).

7. Chang C. Z., Liu M. H., Zhang Z. C., Wang Y. Y., He K., Xue Q. K. Field-effect modulation of anomalous Hall effect in diluted ferromagnetic topological insulator epitaxial films. *Sci. China Phys. Mech.* **59**, 637501 (2016).

8. Yao Y. G., Kleinman L., MacDonald A. H., Sinova J., Jungwirth T., Wang D. S., Wang E. G., Niu Q. First principles calculation of anomalous Hall conductivity in ferromagnetic bcc Fe. *Phys. Rev. Lett.* **92**, 037204 (2004).

9. Yasuda K., Wakatsuki R., Morimoto T., Yoshimi R., Tsukazaki A., Takahashi K. S., Ezawa M., Kawasaki M., Nagaosa N., Tokura Y. Geometric Hall Effects in Topological Insulator Heterostructures. *Nat. Phys.* **12**, 555-559 (2016).
